# Contrasting regulation of the *ecp* common pilus operon in *Citrobacter rodentium*: essential roles of a distal element and IHF even in the absence of H-NS

**DOI:** 10.1007/s00203-026-05216-7

**Published:** 2026-09-21

**Authors:** María I. Isidro-Coxca, Verónica I. Martínez-Santos, Andrés Escalera-Maurer, José L. Puente

**Affiliations:** 1 Departamento de Microbiología Molecular, Instituto de Biotecnología, Universidad Nacional Autónoma de México, Av. Universidad 2001, Cuernavaca, 62210 Morelos México; 2 Present Address: CONACYT-UAGro Universidad Autónoma de Guerrero, Avenida Lázaro 10 Cárdenas s/n, Ciudad Universitaria, Chilpancingo, Guerrero C.P. 39090 México

**Keywords:** Chaperone–usher pathway, Transcriptional regulation, Nucleoid-associated proteins, Integration host factor (IHF), *Cis*-regulatory element, H-NS, Fimbrial gene regulation

## Abstract

The *Escherichia coli* common pilus (ECP) is a chaperone–usher pilus widely distributed in *E. coli* and other enterobacteria, where it mediates adhesion, biofilm formation, and colonization. *Citrobacter rodentium*, a surrogate model for the human attaching-and-effacing pathogens enteropathogenic and enterohemorrhagic *E. coli*, contains a conserved *ecp* operon (*Cr*-*ecp*) whose regulation has substantially diverged from *E. coli*. *Cr-ecp* expression is induced under static DMEM growth at temperatures below 37 °C, driven by a σ⁷⁰-dependent promoter embedded in a regulatory region of approximately 410 bp. We identified an essential distal regulatory element (DRE) between positions − 378 and − 359 relative to the transcription start site, with a 10-bp core required for promoter activity. In contrast to the EcpR-dependent model in *E. coli*, the *ecpR* ortholog is dispensable under the conditions tested. The nucleoid-associated proteins IHF and H-NS bind the regulatory region; IHF is strictly required for activation even when H-NS is absent. These findings define a regulatory architecture distinct from *E. coli ecp*.

**Supplementary Information:**

The online version contains supplementary material available at https://doi.org/10.1007/s00203-026-05216-7.

## Introduction

Initial attachment of intestinal pathogens to host cells is often mediated by fimbriae or pili, multimeric proteinaceous appendages that vary in length and diameter (Berne et al. 2015; Smith and Bharat 2024). These structures mediate bacterial attachment to biotic and abiotic surfaces and frequently constitute the first step in host colonization (Abraham et al. 2015), rendering them attractive therapeutic targets (Psonis and Thanassi 2019).

The *Escherichia coli* common pilus (ECP), also known as the meningitis-associated and temperature-regulated (Mat) fimbria, is a peritrichous fibrillar structure assembled by the chaperone–usher pathway and encoded by the *ecpRABCDE* (*matABCDEF*) operon (Garnett et al. 2012; Pouttu et al. 2001; Rendon et al. 2007; Werneburg and Thanassi 2018). The operon is widely distributed and highly conserved among pathogenic and commensal *E. coli* strains (Burgaya et al. 2023; Munhoz et al. 2018; Rendon et al. 2007; Wurpel et al. 2013). ECP mediates attachment to cultured epithelial cells (Rendon et al. 2007; Saldana et al. 2009) and to extracellular matrix glycoproteins (Mondal et al. 2022; Tsugami et al. 2024), contributes to biofilm formation (Lehti et al. 2010; Yoshida et al. 2022) and to attachment to plant tissues (Rossez et al. 2014; Saldana et al. 2011), and promotes intestinal colonization in infant-mouse and zebrafish models (Munhoz et al. 2023).

Under laboratory conditions, particularly in Dulbecco’s modified Eagle’s medium (DMEM), *ecp* operon expression in enteropathogenic *E. coli* (EPEC) and enterohaemorrhagic *E. coli* (EHEC) is activated by EcpR (also known as MatA), a LuxR-type transcriptional regulator with a C-terminal helix–turn–helix (HTH) DNA-binding domain encoded by the first gene of the operon (Lehti et al. 2013; Martinez-Santos et al. 2012). EcpR binds two distal TTCCT boxes and, together with IHF and RcsB, relieves H-NS-mediated repression of the *ecp* promoter, thereby initiating a positive autoregulatory loop (Martinez-Santos et al. 2012; Pannen et al. 2016). In EHEC, *ecp* expression is further upregulated by acid stress (House et al. 2009) and by EutR in response to ethanolamine (Gonyar and Kendall 2014). The RNA-binding protein Hfq also modulates *ecp* expression (Pearl Mizrahi et al. 2021).

*Citrobacter rodentium* is a mouse-restricted pathogen, closely related to EPEC and EHEC, that causes transmissible colonic crypt hyperplasia (Collins et al. 2014; Mullineaux-Sanders et al. 2019). Although the gene organization of the *ecp* operon is conserved between *C. rodentium* and *E. coli*, the encoded proteins show varying degrees of sequence divergence, and the *ecpR* ortholog is the least conserved. In addition, the *C. rodentium ecp* upstream regulatory region (hereafter *Cr*-*ecp*) shows little sequence conservation with its *E. coli* counterpart (Fig**. **S1). These differences suggested that the regulatory mechanisms controlling ECP expression have also diversified. We therefore used *Cr*-*ecp* as a comparative system to test whether conservation of fimbrial gene organization is accompanied by conservation of regulatory control.

Here, we show that, although some regulatory features are conserved, the *C. rodentium* EcpR ortholog is dispensable for *Cr-ecp* transcription under the conditions tested. Instead, *Cr-ecp* expression depends on an essential distal regulatory element absent from *E. coli* and on the nucleoid-associated protein IHF, which is required for promoter activation, whereas H-NS represses promoter activity. These findings reveal a regulatory architecture distinct from the EcpR-dependent mechanism described in *E. coli*.

## Materials and methods

### Bacterial strains, plasmids and growth conditions

The bacterial strains and plasmids used in this study are described in Table S1. The wild-type strain used throughout this study was *Citrobacter rodentium* DBS100 (also referred to as ICC168; (Petty et al. 2010; Schauer and Falkow 1993)). Plasmids were maintained in *E. coli* DH5α for routine propagation. Unless otherwise specified, bacteria were grown overnight in Luria–Bertani (LB) medium and subcultured for 14 h at 30 °C under static conditions in test tubes containing either LB or Dulbecco’s modified Eagle’s medium (DMEM; Gibco, Thermo Fisher Scientific). When required, antibiotics were added at the following concentrations: ampicillin (Amp), 200 µg/ml; kanamycin (Km), 70 µg/ml; tetracycline (Tc), 10 µg/ml; and chloramphenicol (Cm), 30 µg/ml.

### Molecular biology techniques

Standard procedures were used for genomic and plasmid DNA purification, polymerase chain reaction (PCR), restriction digestion, ligation, transformation, and gel electrophoresis (Green and Sambrook 2012). All restriction and DNA-modifying enzymes were purchased from Thermo Fisher Scientific (Waltham, MA, USA) and used according to the manufacturer’s instructions. Oligonucleotide synthesis and DNA sequencing were performed at the Instituto de Biotecnología, UNAM (Cuernavaca, Mexico).

### Construction of *cat* transcriptional fusions

Oligonucleotides (Table S2) were designed to PCR-amplify fragments spanning different portions of the *Cr-ecp* regulatory region and 5′ coding sequence, using genomic DNA from WT *C. rodentium* as template. The resulting amplicons were purified using the High Pure PCR Product Purification Kit (Roche). Purified fragments were digested with BamHI and HindIII, separated by agarose gel electrophoresis, and extracted from the gel using the Zymoclean Gel DNA Recovery Kit (Zymo Research). The digested fragments were then ligated with T4 DNA ligase (Thermo Fisher Scientific) into plasmid pKK232-8 (Brosius 1984) (Table S1), previously cut with the same enzymes. pKK232-8 carries a promoterless *cat* gene, enabling the construction of transcriptional fusions. The coordinates of each fusion, relative to the *Cr-ecp* transcriptional start site (TSS), are summarized in Table S1 and shown in Figs. 3C and 4A. DRE sequence variants were generated by PCR using forward oligonucleotides bearing the desired base substitutions (Table S2), while maintaining the same 5′ and 3′ coordinates as in p*ecpA*-4.1. All resulting plasmids were verified by sequencing and introduced into *C. rodentium* by electroporation.

### Generation of mutant strains and epitope tagging of chromosomal genes

Mutant strains (Table S1) were generated using the λ Red-based one-step gene inactivation method (Datsenko and Wanner 2000), with primers listed in Table S2. Briefly, *C. rodentium* strains harboring plasmid pKD46 were grown in LB containing L-arabinose at 30 °C to induce the λ Red recombinase, made electrocompetent, and electroporated with PCR products amplified from pKD3 (chloramphenicol resistance cassette) or pKD4 (kanamycin resistance cassette) as templates. Mutants were selected on LB agar plates containing the appropriate antibiotic, and the mutations were verified by PCR using gene-flanking oligonucleotides paired with primers internal to the resistance cassettes: K1 and K2 for the Km cassette, and Cat1 and Cat2 for the Cm cassette (Table S2). After mutagenesis, pKD46 was cured by growth at 37 °C, and its loss was confirmed by sensitivity to ampicillin. The Δ*hns* mutant has been previously described (Caballero-Flores et al. 2015).

To generate strains expressing chromosomally encoded C-terminally 3xFLAG-tagged proteins, we used the one-step gene inactivation method as described by Uzzau et al. (2001). In this case, plasmid pSUB11, which carries the 3xFLAG epitope coding sequence followed by a kanamycin resistance cassette, was used as the PCR template (Uzzau et al. 2001), with the primers listed in Table S2. The resulting PCR product was integrated immediately upstream of the stop codon of the target gene by λ Red-mediated recombination. Successful tagging was verified by PCR using primers flanking the integration site and by western blot detection of the FLAG-tagged protein (see below). To construct the Δ*ecpR ecpA-*3xFLAG::Km strain, the Km resistance cassette was first excised from the Δ*ecpR*::Km mutant by transformation with plasmid pCP20, which encodes the FLP recombinase that acts on the flanking FRT sites. Following selection and confirmation of cassette excision by PCR and antibiotic sensitivity, *ecpA* was subsequently tagged with the 3xFLAG epitope as described above.

### Construction of pT3-*ihfA* and source of complementation plasmids

To construct pT3-*ihfA*, the *ihfA* coding sequence (corresponding to the α subunit of IHF) was PCR-amplified from genomic DNA of EPEC strain E2348/69 using primers ihfA-F and ihfA-R (Table S2). The forward primer carried a sequence encoding an N-terminal 6×His tag in-frame with the *ihfA* coding sequence. The PCR product was digested with BamHI and HindIII and ligated into pMPM-T3 (Mayer 1995) (Table S1), previously cut with the same enzymes, using T4 DNA ligase. The construct was verified by DNA sequencing. Because IHF functions as an αβ heterodimer, complementation of the Δ*ihfA* mutant with pT3-*ihfA* alone restores the IHF holoenzyme, as *ihfB* remains intact in this background and encodes the β subunit. Plasmid pT3-*hns*, used for complementation of the Δ*hns* mutant, has been described previously (Bustamante et al. 2001) (Table S1).

### Chloramphenicol acetyltransferase (CAT) assay

CAT specific activity was determined as previously described (Martinez-Laguna et al. 1999). To obtain whole-cell extracts, pellets from 1.5 ml of bacterial cultures were washed with 800 µl of lysis buffer (50 mM Tris-HCl, pH 7.8, 30 mM DL-dithiothreitol; hereafter TDTT buffer) and resuspended in 300 µl of the same buffer. Samples were sonicated and centrifuged to remove debris and intact cells. CAT activity was assayed in duplicate in 96-well microplates by adding 5 µl of each cell extract to 200 µl of reaction mixture containing 0.1 M Tris-HCl (pH 7.8), 1 mM DTNB [5,5′-dithiobis(2-nitrobenzoic acid); Sigma-Aldrich], 0.1 mM acetyl-CoA (Calbiochem-Merck), and 0.1 mM chloramphenicol (Sigma-Aldrich). Absorbance changes at 410 nm were monitored for 5 min in kinetic mode using a microplate reader (BioTek Instruments, Winooski, VT, USA). Protein concentrations were determined using the BCA Protein Assay Kit (Pierce, Thermo Fisher Scientific). Data are expressed as µmol·min⁻¹·mg⁻¹ of protein and represent the mean ± SD of at least three independent biological replicates, each measured in technical duplicate. CAT activity at 14 h of incubation in LB or DMEM was representative of the maximal activity observed during the growth curve.

### Western blot analysis

Unless otherwise specified, pellets from 3 ml of cultures grown under inducing (DMEM at 30 °C, static) or non-inducing (LB) conditions, as described above, were resuspended in 300 µl of 1× phosphate-buffered saline (PBS) and disrupted by sonication. Samples were mixed with 100 µl of 4× SDS loading buffer, boiled for 10 min, and equal volumes were resolved on 12% (w/v) SDS-PAGE gels. Proteins were transferred onto 0.22-µm pore-size nitrocellulose membranes (Amersham, Cytiva). Transfer efficiency was verified by staining membranes with Ponceau S solution (Sigma-Aldrich) and rinsing with Milli-Q water. Membranes were blocked with 5% (w/v) non-fat dry milk in PBS containing 0.1% Tween-20 (PBS-T) for at least 1 h at room temperature, and incubated with the indicated primary antibody diluted in blocking solution overnight at 4 °C: anti-FLAG M2 (Sigma-Aldrich, F1804, 1:3,000), anti-c-Myc (Sigma-Aldrich, 1:5,000), anti-DnaK (Abcam, 1:10,000), or anti-GroEL (Sigma-Aldrich, 1:50,000). After three washes with PBS-T, membranes were incubated with horseradish peroxidase (HRP)-conjugated goat anti-mouse (Sigma-Aldrich, 1:10,000) or anti-rabbit (Rockland, 1:10,000) secondary antibodies for 1 h at room temperature. Detection was performed using Western Lightning Plus-ECL chemiluminescence reagents (PerkinElmer) according to the manufacturer’s instructions, and signals were captured on Carestream X-OMAT LS autoradiography films (Carestream Health). Western blot experiments were performed using at least three independent biological replicates; representative blots are shown.

### H-NS-MH protein purification

The H-NS-Myc-6×His protein (H-NS-MH) was expressed and purified as described previously (Banda et al. 2018). Briefly, *E. coli* BL21(DE3)pLysS carrying plasmid pMD-HNS-MH (Table S1) was grown to mid-logarithmic phase (OD₆₀₀ ≈ 0.5), and L-arabinose (Sigma-Aldrich) was added to a final concentration of 0.1% (w/v). Cultures were incubated for 4 h at 37 °C. Cells were harvested by centrifugation, resuspended in urea buffer (8 M urea, 100 mM NaH₂PO₄, 10 mM Tris-HCl, pH 8.0), and lysed by sonication. The lysate was centrifuged, and the supernatant was applied to a Ni-NTA agarose column (Qiagen). The column was washed sequentially with urea buffer at pH 8.0 and pH 6.0, and the bound protein was eluted with urea buffer at pH 4.5. Fractions containing purified H-NS-MH were identified by 12% SDS-PAGE. Selected fractions were dialyzed at 4 °C using a Slide-A-Lyzer 10 K MWCO cassette (Thermo Fisher Scientific) against a stepwise gradient of decreasing urea concentrations (4 M, 1 M, and 0.2 M; buffer changes every hour) in 50 mM Tris-HCl (pH 7.5), 10 mM MgCl₂, 20% (v/v) glycerol, 0.5 M NaCl, and 0.1% (v/v) Triton X-100. Final dialysis was performed in H-NS storage buffer containing 30 mM Tris-HCl (pH 7.5), 10 mM MgCl₂, 20% (v/v) glycerol, 240 mM NaCl, 0.1% (v/v) Triton X-100, and 3 mM EDTA. Aliquots were stored at − 70 °C. Protein concentration was determined using the Bradford assay (Bradford 1976).

### Electrophoretic mobility shift assays (EMSAs)

The minimal regulatory region contained in plasmid p*ecpA*-4.1 was amplified by PCR using oligonucleotides EcpABH1Fw-383 and CrecpA-H3R (Table S2). As a negative control, a DNA fragment corresponding to the coding region of the unrelated gene *ROD_27771* was amplified using primers listed in Table S2. Both DNA fragments were purified using the Gel/PCR Extraction Kit (Biomiga) and quantified with a NanoDrop 2000 spectrophotometer (Thermo Fisher Scientific). Approximately 50 ng of each DNA fragment was incubated with increasing concentrations of purified H-NS-MH or IHF (kindly provided by A. Segall, San Diego State University; Rajeev et al. 2007) in binding buffer containing 40 mM HEPES (pH 8.2), 8 mM MgCl₂, 50 mM KCl, 1 mM dithiothreitol (DTT), 0.05% (v/v) Nonidet P-40, and 0.1 mg/ml bovine serum albumin (BSA). Reactions were incubated for 20 min at room temperature and resolved by electrophoresis on 5.5% (w/v) polyacrylamide gels in 0.5× Tris-borate-EDTA (TBE) buffer at 80 V for 3.5 h at 4 °C. Gels were stained with 0.5 µg/ml ethidium bromide and visualized under UV light using a Gel Doc XR+ system (Bio-Rad). Protein concentrations are indicated in the corresponding figure legends. All EMSAs were performed at least three times; representative gels are shown.

### Primer extension analysis

Total RNA was isolated using the RNeasy kit (Qiagen) according to the manufacturer’s instructions from *C. rodentium* WT carrying the p*ecpA*-1 transcriptional fusion grown under inducing conditions. RNA integrity was verified by agarose gel electrophoresis, and concentration was determined using a NanoDrop 2000 spectrophotometer (Thermo Fisher Scientific). Primer extension was performed as described previously (Martinez-Laguna et al. 1999). Briefly, oligonucleotide ecpAHdR + 10 (Table S2), complementary to the *C. rodentium ecpA* coding region (position + 10 relative to the translational start codon), was 5’-end-labeled with T4 polynucleotide kinase (T4 PNK; Thermo Fisher Scientific) and [γ-³²P]ATP (3000 Ci/mmol; PerkinElmer). The labeled primer was hybridized with 15 µg of total RNA, and reverse transcription was carried out for 90 min at 37 °C using M-MLV reverse transcriptase (Thermo Fisher Scientific). A sequencing ladder was generated in parallel using the Thermo Sequenase Kit (USB Corporation; now Thermo Fisher Scientific) with the same labeled oligonucleotide and plasmid p*ecpA*-1 as template, according to the manufacturer’s instructions. Reaction products were resolved on an 8% (w/v) polyacrylamide–7 M urea sequencing gel and visualized by autoradiography on Carestream X-OMAT LS films (Carestream Health). Primer extension experiments were performed at least twice with independent RNA preparations, yielding consistent results.

### Sequence analysis

Nucleotide and amino acid sequences of the *ecp* operon and its upstream regulatory region were retrieved from the Kyoto Encyclopedia of Genes and Genomes (KEGG) database (Kanehisa et al. 2023) for *C. rodentium* DBS100 (also referred to as ICC168; KEGG organism code: cro) and EPEC O127:H6 E2348/69 (KEGG organism code: ecg). The EcpR orthologs correspond to entries ROD_29261 (*C. rodentium*) and E2348C_0250 (EPEC). The *ecpABCDE* genes correspond to ROD_29201 to ROD_29241 in *C. rodentium* and to E2348C_0249 to E2348C_0245 in EPEC. Sequence alignments were performed using Clustal Omega (Sievers et al. 2011) implemented in the EMBL-EBI Job Dispatcher (Madeira et al. 2024); pairwise sequence identities were obtained from the Clustal Omega percent identity matrix. Alignment outputs were exported from the platform and formatted for figure presentation in Microsoft PowerPoint (Microsoft Corporation, Redmond, WA, USA) without modification of the underlying alignment.

To assess conservation of the DRE among *Citrobacter* species, the *ecpA* upstream regulatory region was identified in genome assemblies of *C. freundii* (strain CFNIH1, entry CFNIH1_11140), *C. arsenatis* (strain LY-1, entry E1B03_05980), *C. portucalensis* (strain FDAARGOS_617, entry FOB25_06375), *C. youngae* (strain L6, entry CD187_19255), *C. pasteurii* (strain UMH17, entry CUC49_01180), *C. braakii* (strain FDAARGOS_253, entry A6J81_11885), and *C. sedlakii* (strain 3347689_II, entry JY391_04030), retrieved from KEGG. The 57-bp region encompassing the DRE and its immediate flanking sequence was extracted from each orthologous *ecpA* upstream region and aligned using Clustal Omega. Asterisks below the alignment indicate columns of full sequence identity across all species compared, while dots indicate positions conserved in all but one sequence. Additional conserved positions outside the functionally defined DRE were also observed near the end of the alignment and elsewhere along the regulatory region (not shown); their significance remains unknown but may reflect broader evolutionary conservation of the *ecp* regulatory region among *Citrobacter* species. The alignment is presented in Fig. S2.

### Statistical analysis

Quantitative data are presented as mean ± standard deviation (SD) from at least three independent biological replicates, each measured in technical duplicate, unless otherwise indicated. Statistical analyses were performed using IBM SPSS Statistics version 26.0 (IBM Corp., Armonk, NY, USA) and graphical representation using GraphPad Prism version 10.2.3 (GraphPad Software, San Diego, CA, USA). Data distribution was assessed by Shapiro–Wilk test. Because data did not follow normal distribution, non-parametric tests were applied: Kruskal–Wallis test was used to compare multiple groups, followed by Mann–Whitney U tests for pairwise comparisons. Differences were considered statistically significant when *P* < 0.05. Statistical significance is indicated in the figures as follows: *P* < 0.05 (*), *P* < 0.01 (**), and ns, not significant.

## Results

### Comparative sequence analysis reveals divergence of the *ecp* operon and its regulatory region between *C. rodentium* and *E. coli*

**Fig. 1:**
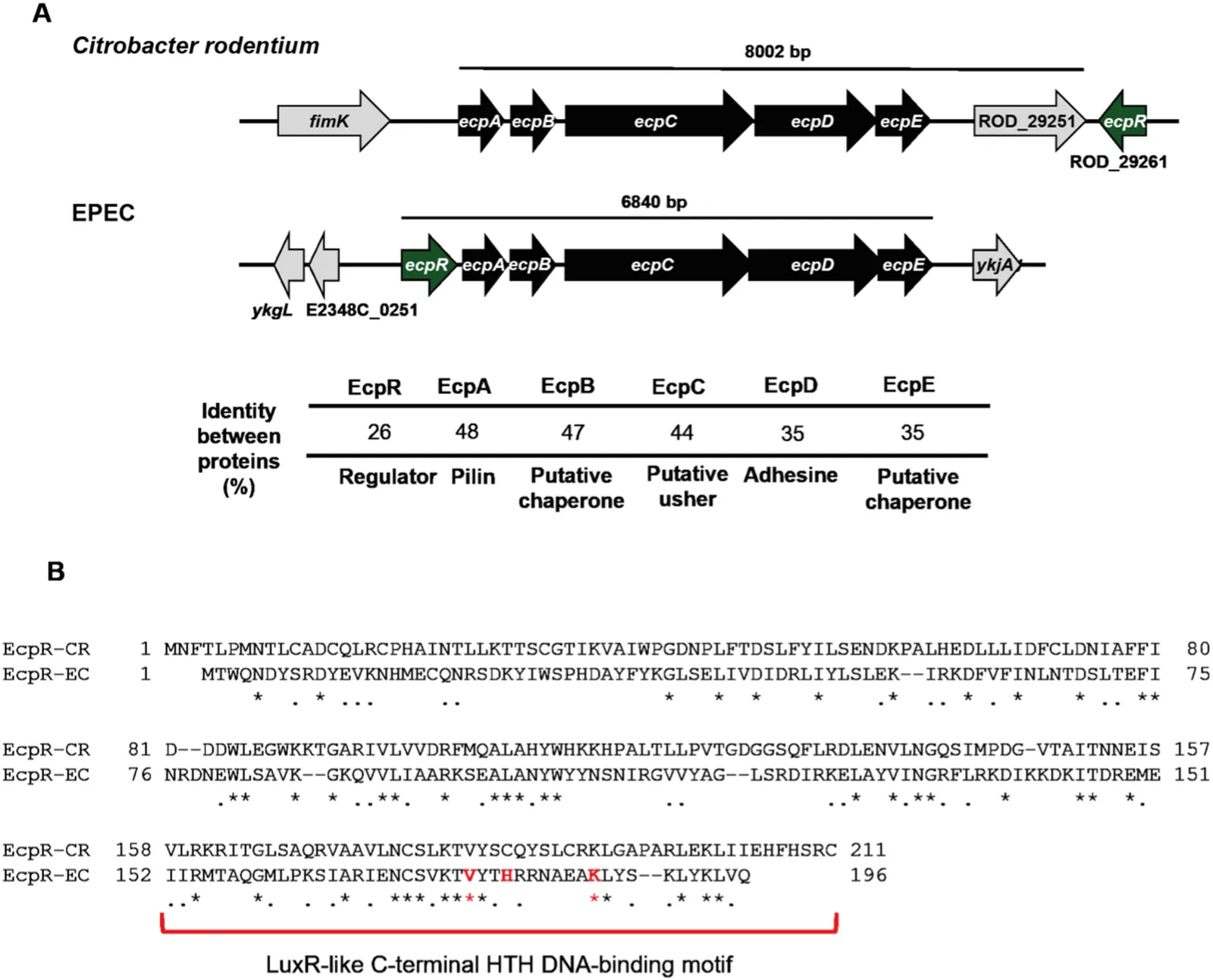
Genomic organization of the *ecp* operon and divergence of EcpR orthologs in *Citrobacter rodentium* and EPEC. **A** Schematic representation of the *ecp* gene clusters in *C. rodentium* ICC168 and EPEC O127:H6 E2348/69. Arrows indicate coding sequences and lines represent intergenic regions. Genes encoding proteins involved in ECP assembly are shown in black, and *ecpR* and its *C. rodentium* ortholog in dark green. Locus tags: ROD_29261 and ROD_29201–ROD_29241 (*C. rodentium*); E2348C_0250 and E2348C_0249–E2348C_0245 (EPEC). The table below the schematic indicates the percentage of amino acid identity between orthologous pairs. **B** Alignment and domain representation of EcpR orthologs from *C. rodentium* and EPEC, highlighting the C-terminal GerE/LuxR-family helix–turn–helix (HTH) DNA-binding domain. Residues V176 and K186 (numbered according to *E. coli* EcpR), retained in the *C. rodentium* ortholog, are indicated; the H179 residue, not conserved in *C. rodentium*, is also marked. Sequences were retrieved from KEGG and aligned with Clustal Omega; pairwise identities were calculated from the Clustal Omega percent identity matrix

In *E. coli*, the *ecp* operon (*ecpRABCDE*) encodes the transcriptional activator EcpR (Martinez-Santos et al. 2012) and five proteins of the chaperone–usher pilus assembly system: the major pilin EcpA, the chaperones EcpB and EcpE, the usher EcpC, and the tip adhesin EcpD (Fig. 1A) (Garnett et al. 2012).

Comparative analysis of the *ecp* operons from *E. coli* and *C. rodentium* revealed conserved gene organization but substantial sequence divergence of the encoded proteins (Fig. 1A). Among the structural proteins, pairwise amino acid identities ranged from 35% (EcpD and EcpE) to 48% (EcpA), with EcpA being the most conserved, followed by EcpB (47%) and EcpC (44%). The EcpR ortholog, the most divergent of the predicted proteins, shares only 26% amino acid identity with its *E. coli* counterpart. Furthermore, its gene is encoded on the complementary strand and located downstream of the *ecpABCDE* cluster, rather than at the start of the operon as in *E. coli*.

Both EcpR orthologs share the same overall domain architecture, characterized by a C-terminal GerE/LuxR-family helix–turn–helix (HTH) DNA-binding domain. However, the modest pairwise identity (26%) and sequence variations within and around the HTH domain suggest substantial divergence that may influence the functionality of the regulator. Within the DNA-binding domain, the *C. rodentium* EcpR ortholog retains residues V176 and K186 (numbered according to *E. coli* EcpR), previously identified as critical for *E. coli* EcpR activity (Martinez-Santos et al. 2012). However, the H179 residue, also reported as essential for *E. coli* EcpR function (Lehti et al. 2013), is not conserved in the *C. rodentium* ortholog (Fig. 1B).

The *Cr-ecp* upstream regulatory region also shows very low sequence identity with its *E. coli* counterpart and lacks the TTCCT boxes recognized by EcpR (Martinez-Santos et al. 2012) (Fig. S1).

Together, these comparative analyses suggested that the regulatory mechanisms controlling *ecp* expression have also diverged between *C. rodentium* and *E. coli*. To investigate this possibility, we first defined the conditions under which *Cr-ecp* is expressed.

### Growth conditions modulate *ecp* expression in *C. rodentium*

In *E. coli*, ECP synthesis is induced in DMEM at temperatures below 37 °C and responds to multiple environmental signals (Lehti et al. 2013; Martinez-Santos et al. 2012; Pouttu et al. 2001; Rendon et al. 2007; Saldana et al. 2011). To define the conditions inducing *Cr*-*ecp* expression, we constructed a transcriptional fusion containing positions − 494 to + 211 relative to the transcriptional start site (mapped in Fig. 3) cloned upstream of the promoterless *cat* gene, generating plasmid p*ecpA*-1 (Table S1; Fig. 3C). Promoter activity was assessed in wild-type *C. rodentium* grown under static or shaken conditions in DMEM or LB.

**Fig. 2:**
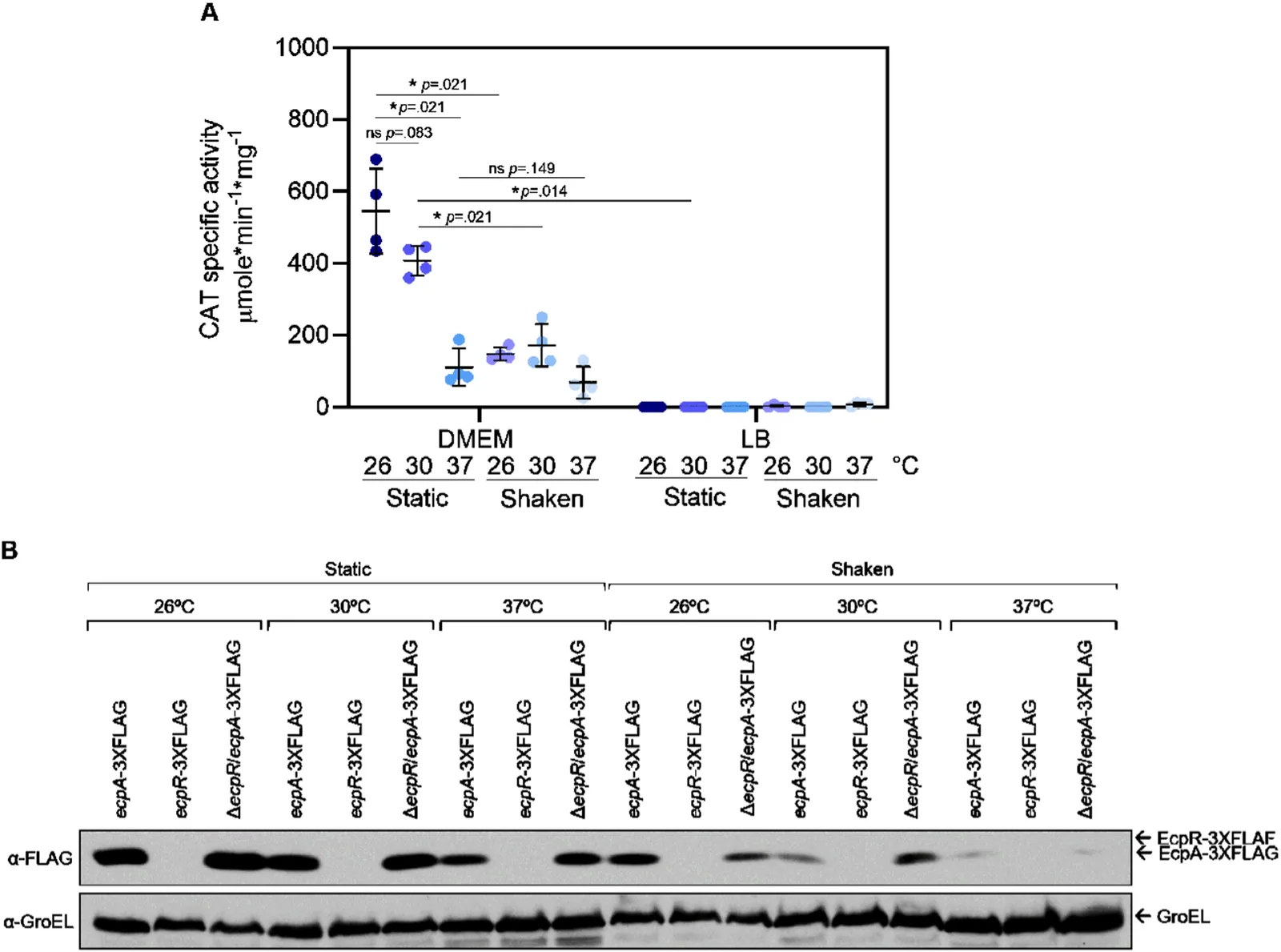
*Cr-ecp* expression is regulated by environmental conditions but does not require EcpR. **A** CAT specific activity of wild-type *C. rodentium* carrying the p*ecpA*-1 transcriptional fusion grown under the indicated conditions. Samples were collected at the time point corresponding to maximal activity (12 h). Data represent mean ± SD of two independent biological replicates, each measured in technical duplicate. Statistical significance was assessed using the Kruskal–Wallis test, followed by Mann–Whitney U tests for pairwise comparisons. *, *P* < 0.05; ns, not significant. **B** Western blot analysis of whole-cell extracts from *C. rodentium* strains carrying chromosomal *ecpA*-3xFLAG or *ecpR*-3xFLAG fusions in wild-type *or* Δ*ecpR* backgrounds, grown in DMEM under static or shaken conditions at the indicated temperatures. EcpA-3xFLAG and EcpR-3xFLAG were detected with anti-FLAG antibodies; GroEL was used as a loading control. Images are representative of at least three independent experiments

CAT activity was highest in static DMEM cultures at temperatures below 37°C, whereas no detectable activity was observed in LB under any condition tested (Fig. 2A). These results are consistent with previous observations in *E. coli* (Lehti et al. 2013; Martinez-Santos et al. 2012). Based on these results, we defined static growth in DMEM at 26°C or 30°C as ‘inducing conditions’ and growth in LB as ‘non-inducing’ for *Cr*-*ecp* expression throughout this study.

To validate these findings at the protein level, the *ecpA* gene was C-terminally tagged with a 3xFLAG epitope sequence at its chromosomal location in wild-type *C. rodentium*, generating strain *ecpA*-3xFLAG (Table S1). This strain was grown in static or shaken DMEM at 26 °C, 30 °C, and 37 °C, and EcpA-3xFLAG levels were analyzed by western blot, with DnaK as loading control. Consistent with the transcriptional fusion data (Fig. 2A), EcpA-3xFLAG levels decreased progressively as temperature increased from 26 °C to 37 °C (Fig. 2B; representative of *n* = 3 independent biological replicates), confirming temperature-dependent regulation of EcpA production. This concordance between the CAT reporter and native, chromosomally expressed EcpA supports the reliability of the transcriptional fusion system used throughout this study.

### The EcpR ortholog is dispensable for *Cr-ecp* activation in *C. rodentium*

In *E. coli*, EcpR positively regulates *ecp* expression (Lehti et al. 2013; Martinez-Santos et al. 2012). In *C. rodentium* the *ecpR* ortholog is located downstream of the *ecpABCDE* cluster on the complementary strand and shares only 26% amino acid identity with its *E. coli* counterpart (Fig. 1).

To assess the role of the *C. rodentium ecpR* ortholog in *Cr-ecp* activation, we deleted this gene in the *ecpA*-3xFLAG background, generating strain Δ*ecpR ecpA*::3XFLAG-Km (Table S1). EcpA-3xFLAG levels were compared between the mutant and the wild-type strain in whole-cell extracts from static and shaken DMEM cultures grown at 26 °C, 30 °C, and 37 °C.

EcpA-3xFLAG was produced at comparable levels in wild-type and Δ*ecpR* strains at 26 °C, and both strains showed a similar decrease at higher temperatures (Fig. 2B; representative of *n* = 3 independent biological replicates). These results indicate that the EcpR ortholog is dispensable for *Cr-ecp* activation under the conditions tested, in contrast to its essential role in *E. coli* (Lehti et al. 2013; Martinez-Santos et al. 2012). An EcpR-3xFLAG fusion expressed from its native chromosomal locus (Table S1) was not detectable by western blot under inducing conditions (Fig. 2B), suggesting that the protein is either expressed at very low levels or is unstable. Nonetheless, the absence of a phenotype in the Δ*ecpR* mutant supports the conclusion that this regulator is not required for *Cr-ecp* activation in this experimental setting.

### Identification of the *Cr-ecp* promoter

The results described above indicated that the *Cr-ecp* upstream region in plasmid p*ecpA*-1 contains an active promoter induced under static growth in DMEM at temperatures below 37 °C (Fig. 2A). To map this promoter and identify its transcriptional start site, we performed primer extension analysis using RNA isolated from wild-type *C. rodentium* carrying p*ecpA*-1 grown under inducing conditions.

**Fig. 3:**
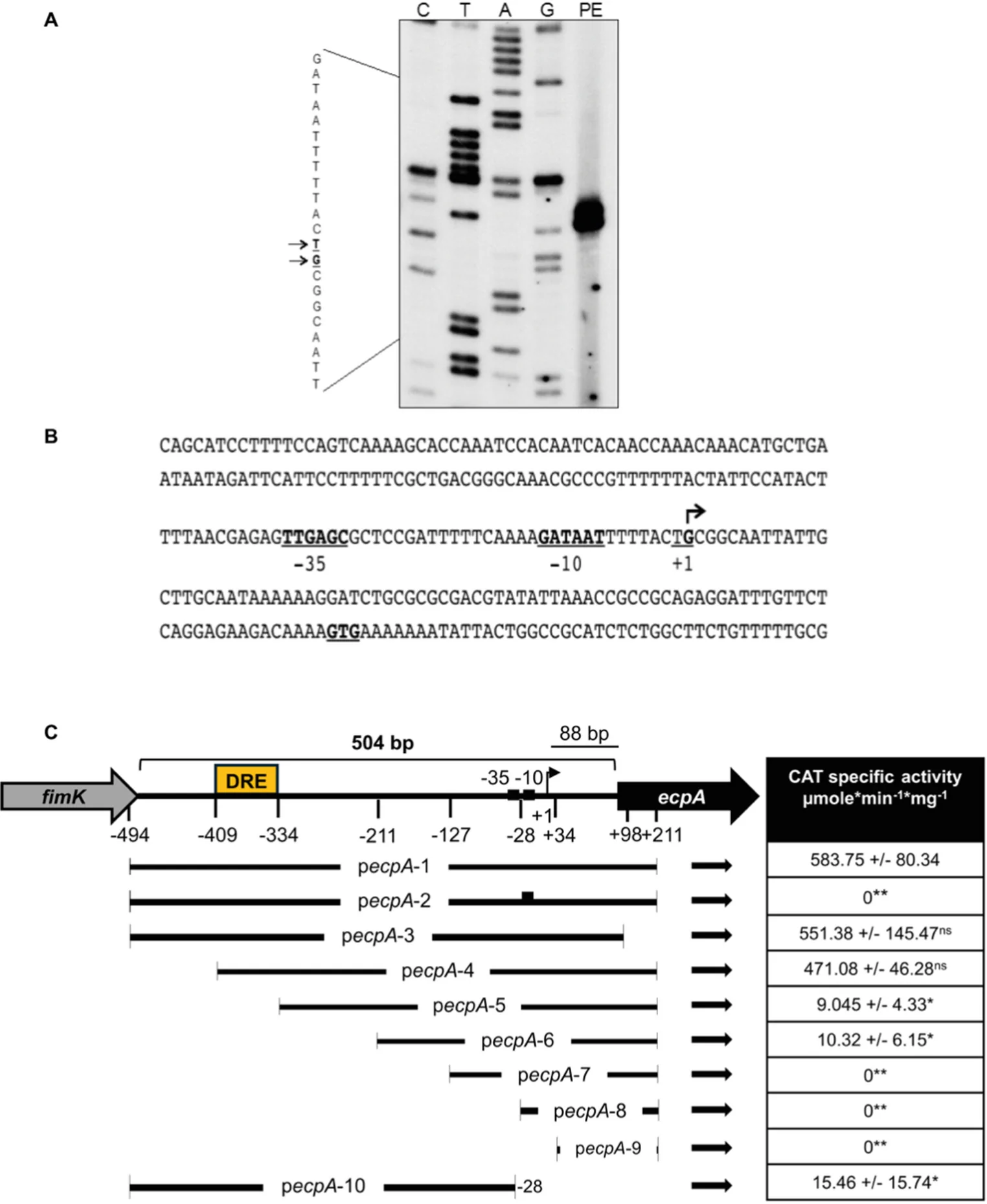
A distal regulatory region is essential for *Cr-ecp* expression. **A** Primer extension analysis identifying the transcription start site of the *Cr-ecp* promoter. Total RNA was isolated from wild-type *C. rodentium* carrying p*ecpA*-1 grown under inducing conditions. Two transcription start sites at a T residue and at the adjacent G residue (designated + 1, position − 88 relative to the *ecpA* start codon) are underlined and indicated by an arrow. Representative of two independent biological replicates, using RNA isolated from bacterial cultures grown on separate days. **B** DNA sequence of the *Cr-ecp* promoter region. The transcription start site (+ 1 and broken arrow), the predicted − 35 (TTGAGC) and − 10 (GATAAT) and the *ecpA* start codon (GTG) are in bold and underlined. **C** Schematic representation of the transcriptional fusions used to delimit the regulatory region (p*ecpA*-1 to p*ecpA*-10; Table S1). The transcription start site is indicated by a broken arrow, the − 35 and − 10 promoter elements by black boxes, and the region containing the distal regulatory element (DRE) by a white rectangle. CAT specific activity was measured in wild-type *C. rodentium* carrying the indicated fusions and grown in static DMEM at 30 °C. Data represent mean ± SD of at least three independent biological replicates, each measured in technical duplicate. Statistical significance was assessed using the Kruskal–Wallis test, followed by Mann–Whitney U tests for pairwise comparisons. *P < 0.05; **P < 0.01; ns, not significant

Primer extension identified two transcription start sites at adjacent T and G residues located 89 and 88 nucleotides upstream of the *ecpA* start codon, respectively (Fig. 3A and B). The G residue at position − 88 was designated as + 1 for subsequent analyses. Based on this assignment, putative − 35 and − 10 promoter elements were identified as TTGAGC and GATAAT, respectively, separated by 17 bp and matching four of six positions in the − 35 consensus (TTGACA) and five of six positions in the − 10 consensus (TATAAT) of σ⁷⁰-dependent promoters (Harley and Reynolds 1987).

To confirm the functionality of the identified − 10 element, the wild-type sequence GATAAT was replaced with the non-consensus sequence TCGCCG (six base substitutions) by overlap PCR using p*ecpA*-1 as template, generating fusion p*ecpA*-2 (Table S1; Fig. 3C). This mutation reduced promoter activity to background levels in wild-type *C. rodentium* under inducing conditions (Fig. 3C; mean ± SD of *n* = 3 independent biological replicates), confirming that the identified − 10 element is essential for promoter function and that this region constitutes the functional *Cr-ecp* promoter.

### The *Cr-ecp* regulatory region contains a distal element required for activation

To define the minimal regulatory region of *Cr-ecp* and identify regulatory elements, we performed a systematic 5′-to-3′ deletion analysis of the region contained in the p*ecpA*-1 transcriptional fusion. This generated a series of *cat* fusions (p*ecpA*-4 to p*ecpA*-9), lacking increasing portions of the upstream sequence (Fig. 3C). CAT activity assays in wild-type *C. rodentium* under inducing conditions revealed that a region spanning positions − 409 to − 334 relative to the transcription start site (+ 1) is required for promoter activity. Fusions p*ecpA*-5 to p*ecpA*-9, which lack this region, showed CAT activity at background levels (Fig. 3C; mean ± SD of n = 3 independent biological replicates), whereas p*ecpA*-4 retained activity comparable to p*ecpA*-1. In addition, fusion p*ecpA*-3, which lacks 110 bp of the *ecpA* 5’ coding region relative to p*ecpA*-1, retained full activity (Fig. 3C), confirming that *ecpA* coding sequences downstream of the promoter are not required for activity.

To determine whether the sequence between positions − 409 and − 334 itself functions as a promoter or as an upstream regulatory element, we constructed fusion p*ecpA*-10, in which all sequences downstream of position − 28, including the σ⁷⁰ −10 element, were deleted. The background activity of p*ecpA*-10 (Fig. 3C) demonstrates that the σ⁷⁰ promoter accounts for most of the activity observed in p*ecpA*-1 and that the − 409 to − 334 region does not function as an independent promoter, but rather as an upstream regulatory element required for *Cr-ecp* transcription.

Together, these results indicate that the minimal regulatory region of *Cr-ecp* extends from position − 409 to the transcription start site, with the σ⁷⁰ promoter located within a relatively long upstream regulatory region of approximately 410 bp. The sequence located between positions − 409 and − 334, essential for *Cr-ecp* expression under the conditions tested, is hereafter referred to as the distal regulatory element (DRE); its minimal functional core is refined in the following section.

### Refinement of the distal regulatory element (DRE)

**Fig. 4:**
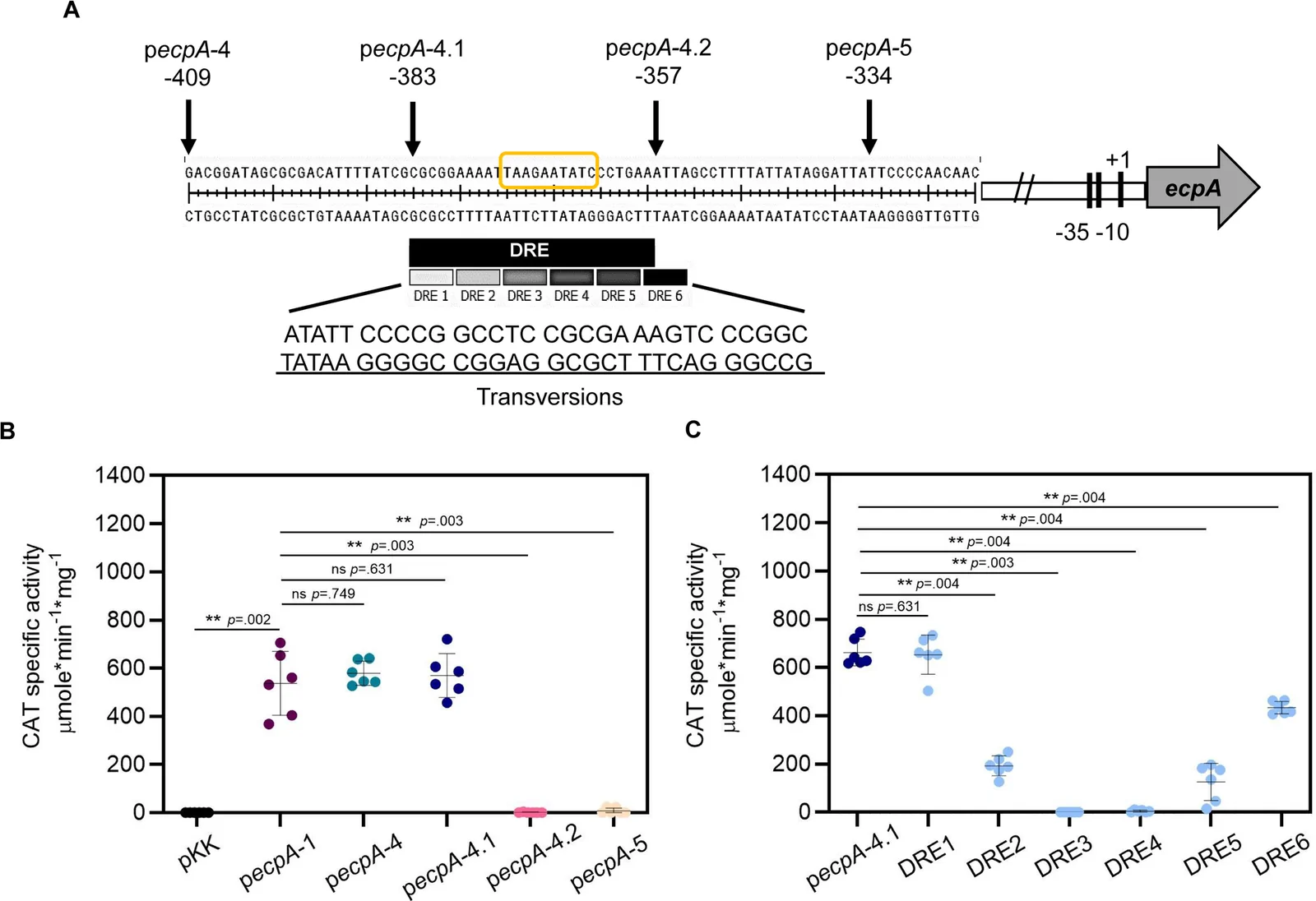
The distal regulatory element (DRE) is located between positions − 378 and − 359 relative to the *Cr-ecp* transcription start site. **A** Schematic representation of the *Cr-ecp* upstream regulatory region indicating the position of the DRE (positions − 378 to − 359), the 10-bp core sequence TAAGAATATC (yellow box), and the 5′ limits of the transcriptional fusions analyzed (p*ecpA*-4, p*ecpA*-4.1, p*ecpA*-4.2, p*ecpA*-5; Table S1). The six 5-bp segments (DRE1–DRE6) subjected to mutational analysis are also indicated. **B** Determination of the minimal regulatory region required for *Cr-ecp* expression. CAT specific activity was measured in wild-type *C. rodentium* carrying fusions p*ecpA*-4, p*ecpA*-4.1, p*ecpA*-4.2, or p*ecpA*-5 (Table S1) and grown in static DMEM at 30 °C. **C** Identification of essential motifs within the DRE. The DRE was subdivided into six 5-bp segments (DRE1–DRE6), each subjected to transversions designed to maximize disruption of protein–DNA contacts. CAT specific activity was measured in wild-type *C. rodentium* carrying the p*ecpA*-4.1 derivative fusions and grown in static DMEM at 30 °C. Segments DRE3 and DRE4 (corresponding to the core sequence TAAGAATATC, red box in panel A) are highlighted. For panels B and C, data represent mean ± SD of three independent biological replicates, each measured in technical duplicate. Statistical significance was assessed using the Kruskal–Wallis test, followed by Mann–Whitney U tests for pairwise comparisons. **P < 0.01; ns, not significant. Comparisons were made to strains carrying p*ecpA*-1 (**B**) or p*ecpA*-4.1 (**C**), as indicated

To delimit the minimal functional sequence within the DRE, we generated two shorter fusions derived from p*ecpA*-4 (Fig. 4A): p*ecpA*-4.1, lacking 26 5’-most bp (positions − 383 to + 211), and p*ecpA*-4.2, lacking 52 bp from the 5’ end (positions − 357 to + 211). We compared the CAT activity of these constructs with that of p*ecpA*-4 and p*ecpA*-5 in wild-type *C. rodentium* under inducing conditions. CAT activity from p*ecpA*-4.1 was comparable to that of p*ecpA*-4, whereas p*ecpA*-4.2 showed no detectable activity, similar to p*ecpA*-5 (Fig. 4B; mean ± SD of n = 3 independent biological replicates). These results delimit the 5’ boundary of the functional DRE to a position between − 383 and − 357.

To further refine the functional limits of the DRE, we introduced 5-bp transversions in successive segments spanning positions − 383 to − 354 within p*ecpA*-4.1, generating the DRE1–DRE6 series of transcriptional fusions (Fig. 4A; segments and exact positions listed in Table S1). Transversions were chosen to maximize disruption of potential protein–DNA contacts. CAT activity assays in wild-type *C. rodentium* under inducing conditions revealed a graded effect across the mutated segments (Fig. 4C; mean ± SD of *n* = 3 independent biological replicates). Substitutions in DRE3 and DRE4 (positions − 373 to − 364) abolished activity, identifying the 10-bp sequence TAAGAATATC as an absolutely required core. Substitutions in DRE2 (positions − 378 to − 374) and DRE5 (positions − 363 to − 359) reduced activity to 20–30% of the wild-type fusion, indicating that the 5-bp segments immediately flanking the core also contribute substantially to DRE function. Substitutions in DRE6 (positions − 358 to − 354) reduced activity to ~ 65% of the wild type, suggesting an additional, modest contribution from this distal segment. Substitutions in DRE1 (positions − 383 to − 379) did not affect activity (~ 100% of the wild type), consistent with the full activity retained by p*ecpA*-4.1.

Together, these results define the DRE as a ~ 20-bp element spanning positions − 378 to − 359, with TAAGAATATC as the essential core and flanking segments contributing additional regulatory information.

To explore whether the DRE is conserved beyond *C. rodentium* in the *Citrobacter* genus, we compared the DRE region with orthologous *ecpA* upstream sequences from seven additional *Citrobacter* species (Fig. S2). The 10-bp core sequence corresponding to DRE3 and DRE4, the segments essential for promoter activation (Fig. 4C), was among the most highly conserved positions across the alignment, whereas conservation was comparatively lower in the flanking DRE1, DRE2, DRE5, and DRE6 segments and in *C. sedlakii*, the most divergent species examined. This pattern suggests that the functional core of the DRE may be conserved across *Citrobacter* species, although its role in these species has not been experimentally tested.

### IHF is required for *Cr-ecp* activation independently of H-NS-mediated repression

H-NS (histone-like nucleoid structuring protein) and IHF (integration host factor) are nucleoid-associated proteins (NAPs) that frequently exert opposing effects on transcription, with H-NS silencing AT-rich or horizontally acquired loci and IHF promoting DNA bending and long-range regulatory interactions (Dillon and Dorman 2010; Khrapunov et al. 2006; Rashid and Dame 2024).

**Fig. 5:**
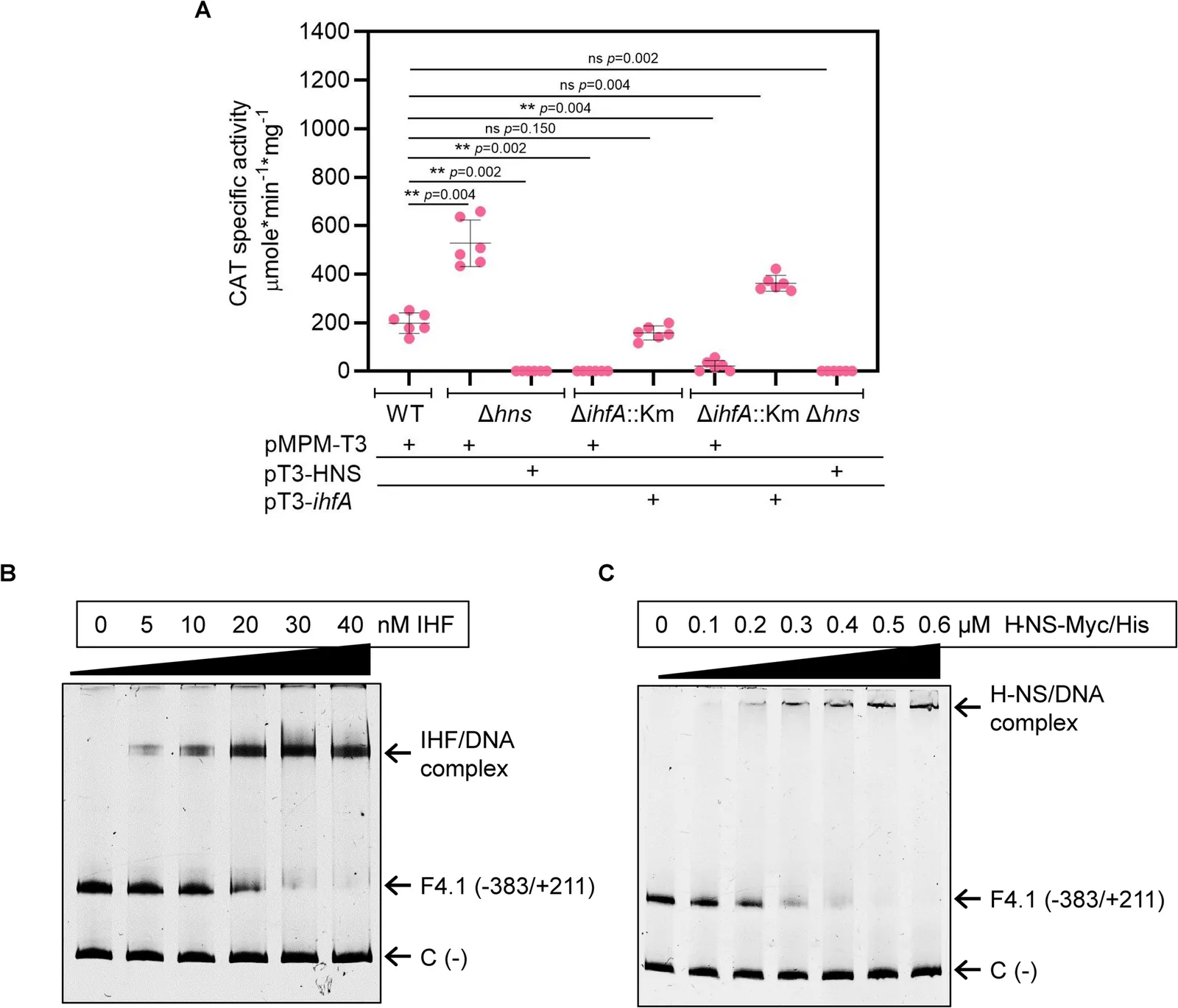
Nucleoid-associated proteins H-NS and IHF negatively and positively regulate *Cr-ecp* expression, respectively. **A** CAT specific activity of p*ecpA*-1 in wild-type *C. rodentium* and in Δ*hns*, Δ*ihfA*::Km, and Δ*hns* Δ*ihfA*::Km double mutant strains. Mutant strains were complemented with plasmids pT3-*hns* or pT3-*ihfA* (Table S1) as indicated; control strains carry the empty vector pMPM-T3. Cultures were grown in static DMEM at 30 °C. Data represent mean ± SD of three independent biological replicates, each measured in technical duplicate. Statistical significance was assessed using the Kruskal–Wallis test, followed by Mann–Whitney U tests for pairwise comparisons; comparisons were made to the wild-type strain. **P < 0.01; ns,* not significant.* **B**, **C** Electrophoretic mobility shift assays showing binding of purified IHF (B) and H-NS-MH (C) to the *Cr-ecp* regulatory fragment from p*ecpA*-4.1. Increasing protein concentrations (0 to 40 nM for IHF; 0 to 600 nM for H-NS-MH) were incubated with approximately 50 ng of the indicated DNA fragment under the conditions described in Materials and Methods. A DNA fragment corresponding to *ROD_27771* (an unrelated chromosomal locus) was used as a negative control. Representative images from at least three independent experiments are shown

In EPEC and EHEC, H-NS represses and IHF activates *ecp* expression (Martinez-Santos et al. 2012). Because H-NS and IHF are highly conserved between *C. rodentium* and *E. coli*, we examined the role of these proteins in *Cr-ecp* regulation. CAT activity of p*ecpA*-1 was measured in wild-type *C. rodentium* and in Δ*hns* or Δ*ihfA*::Km mutants, each carrying the empty vector pMPM-T3 (Table S1) as a control for plasmid-related effects. CAT activity from p*ecpA*-1 increased approximately 2.5X-fold in the Δ*hns* mutant and was abolished in the Δ*ihfA*::Km mutant (Fig. 5A). Both phenotypes were restored upon complementation with plasmids pT3-*hns* or pT3-*ihfA*, respectively (Fig. 5A).

To test whether the requirement for IHF simply reflects its role in counteracting H-NS-mediated repression, we examined p*ecpA*-1 activity in a Δ*hns* Δ*ihfA*::Km double mutant. CAT activity was not detected in this background (Fig. 5A), indicating that IHF is essential for *Cr-ecp* expression even in the absence of H-NS.

To determine whether H-NS and IHF act directly on the *Cr-ecp* regulatory region, we performed EMSAs using purified H-NS-MH or IHF and the regulatory fragment contained in the p*ecpA*-4.1 transcriptional fusion, spanning positions − 383 to + 211 relative to the *Cr-ecp* transcriptional start site. Both H-NS-MH and IHF formed protein–DNA complexes with the *Cr-ecp* regulatory fragment in a concentration-dependent manner (Fig. 5B and C), whereas a control DNA fragment corresponding to the unrelated gene *ROD_27771* remained unshifted at all protein concentrations tested.

Candidate binding sites within the 500 bp upstream of *ecpA* were identified using the Virtual Footprint platform (https://www.prodoric.de/vfp/) with the *E. coli* position weight matrix for IHF. The IHF consensus sequence (5′-WATCAANNNNTTR-3′) is highly degenerate, a feature thought to underlie the mechanistic versatility of IHF: rather than acting through strict sequence recognition, IHF induces a pronounced DNA bend of approximately 160°, and its regulatory function typically depends on this local architecture, bringing activators into proximity, promoting DNA looping, stabilizing nucleoprotein complexes, or facilitating or hindering RNA polymerase access (Dillon and Dorman 2010; Khrapunov et al. 2006). Consistent with this degeneracy, the search returned multiple candidate sites across the analyzed region; we therefore report only those with the highest relative score (0.90–0.95). One such site is located immediately downstream of the DRE, while two additional candidate sites overlap within 65 bp downstream of the DRE3-DRE4 core, two on the negative strand and one on the positive strand (Fig. S1). A similar strategy was used to search for candidate H-NS binding sites, identifying three sequences as putative binding sites expected to function as nucleation sites. It should be emphasized that these are computational predictions based on sequence similarity to known consensus motifs and do not constitute direct evidence of protein occupancy at these positions. These sites lie closer to the DRE, raising the interesting possibility that H-NS may interfere with *Cr*-*ecp* transcription by disrupting the binding of regulatory elements that interact with the DRE, rather than by directly occluding the promoter itself.

Nonetheless, establishing the functional relevance of these predicted sites will require future footprinting and fine-scale EMSA mapping, followed by site-directed mutagenesis of those sites with the strongest experimental support. This is particularly important given that we cannot rule out a contribution from other predicted sites for either regulator that scored lower in this analysis.

Together, these results demonstrate that H-NS binds directly to the *Cr-ecp* regulatory region in vitro and is required for full repression of promoter activity in vivo, whereas IHF binds the same region and is essential for its activation through a mechanism that extends beyond relief of H-NS-mediated silencing. The in silico-predicted binding sites described above provide candidate locations consistent with this activity, but do not by themselves establish the specific protein–DNA contacts underlying these functions, which remain to be defined experimentally. This suggests that IHF helps establish a promoter architecture required for transcriptional activation, possibly in coordination with the DRE and additional trans-acting regulators that remain to be defined.

## Discussion

Most enterobacteria encode multiple fimbrial operons whose individual contributions to virulence and colonization remain incompletely characterized, in part because the regulatory logic governing selective expression of each locus is poorly understood. In this work, we addressed this gap by characterizing the regulatory architecture of the *C. rodentium ecp* operon (*Cr-ecp*) and found that it differs substantially from the EcpR-dependent model described in *E. coli* (Lehti et al. 2013; Martinez-Santos et al. 2012). Several features of the *Cr-ecp* locus suggested that the *E. coli* model might not apply: the divergence of the upstream regulatory region (Fig. S1), the absence of the EcpR-binding TTCCT boxes, the altered genomic position of the *ecpR* ortholog, and its low sequence conservation. Consistent with this, the *C. rodentium ecpR* ortholog proved dispensable for *Cr-ecp* activation under the conditions tested, in contrast to the essential role of EcpR in *E. coli*.

A defining feature of *Cr-ecp* is the presence of an extended upstream regulatory region containing a distal *cis*-acting element (DRE) essential for transcriptional activation under the conditions examined in this study, organized around a 10-bp core sequence (TAAGAATATC) with flanking nucleotides contributing additional regulatory information (Fig. 4). Comparative analysis across *Citrobacter* species further indicates that the DRE core (DRE3-DRE4) is preferentially conserved relative to its flanking sequence (Fig. S2), consistent with this core representing the primary determinant of DRE function identified in *C. rodentium*. However, it should be noted that sequence conservation does not by itself demonstrate conservation of regulatory function; whether the DRE core is bound by an equivalent trans-acting factor and drives *ecp* expression in these other *Citrobacter* species remains to be experimentally determined.

Such long-range regulatory configurations have been described in bacterial promoters controlled by DNA-bending proteins, nucleoid-associated proteins (NAPs), or enhancer-like activators (Busby and Browning 2024; Ge et al. 2025; Hustmyer and Landick 2024). Based on its characteristics with respect to the transcriptional start site, the *Cr-ecp* promoter is σ⁷⁰-dependent, suggesting that the DRE operates differently from σ⁵⁴-dependent bacterial enhancers. A relevant analogy is provided by VirB of *Shigella* spp., which mediates long-range alleviation of H-NS repression at virulence loci (Cris et al. 2025; Weatherspoon-Griffin et al. 2018); however, in contrast to its essential role in *Cr-ecp*, IHF is not required for VirB-dependent alleviation.

**Fig. 6:**
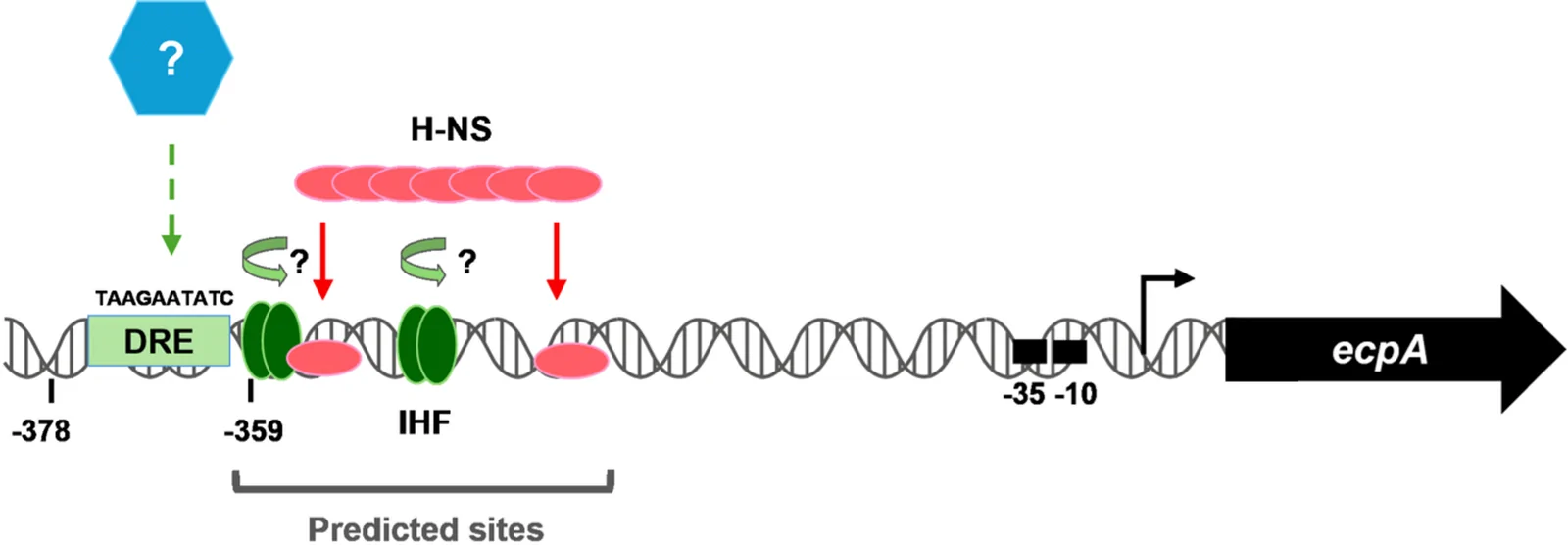
Proposed regulatory model for transcriptional activation of the *Citrobacter rodentium ecp* fimbrial operon. This model illustrates one possible interpretation of the genetic requirements and binding activities described in this study and should not be regarded as an experimentally validated regulatory pathway. Under inducing conditions (static growth in DMEM at temperatures below 37 °C), *Cr-ecp* expression is controlled by an extended upstream regulatory region of approximately 410 bp containing a distal regulatory element (DRE) essential for transcriptional activation. The nucleoid-associated protein IHF binds directly to the *Cr*-*ecp* regulatory region (Fig. 5) and is proposed to introduce DNA bending that may bring the DRE into close spatial proximity with the σ⁷⁰ RNA polymerase holoenzyme bound at the promoter, although this has not been directly demonstrated. Additional trans-acting regulatory inputs at the DRE remain to be characterized. The nucleoid-associated protein H-NS also binds directly to the regulatory region and represses promoter activity; however, IHF-dependent activation is required even in the absence of H-NS, indicating that IHF provides a positive function distinct from simple H-NS antagonism, one that we hypothesize is architectural in nature, although this remains to be formally established. This regulatory model differs from the EcpR-dependent activation described for the *ecp* operon in EPEC and EHEC. The schematic shows potential binding sites for IHF (green ovals) and for H-NS (light red ovals), the DRE (light green rectangle showing the DRE3 and DRE4 essential sequence motif, Fig. 4), and the putative unknown *trans*-acting element (blue hexagon), which remains to be identified. Solid shapes indicate proteins with demonstrated binding to the *ecpA* upstream region (EMSA, Fig. 5); the specific positions shown (*Predicted sites*) are computationally predicted and have not been experimentally validated

This requirement for IHF raises the possibility that higher-order DNA architecture contributes to *Cr-ecp* activation (Fig. 6). IHF is a nucleoid-associated heterodimer that introduces sharp DNA bends and contributes to transcriptional regulation by bringing distant DNA segments into close spatial proximity (Dillon and Dorman 2010; Khrapunov et al. 2006). The strict requirement for IHF in *Cr-ecp*, preserved even in the absence of H-NS (Fig. 5A), indicates that its role cannot be explained by simple relief of H-NS-mediated silencing. This distinguishes *Cr-ecp* regulation from the model described for EPEC and EHEC, in which IHF primarily facilitates EcpR-dependent antisilencing and becomes largely dispensable when H-NS repression is removed (Martinez-Santos et al. 2012). In *C. rodentium*, IHF therefore appears to provide a positive function in promoter activation that extends beyond antisilencing, one that we hypothesize is architectural in nature, although this remains to be formally established.

H-NS also participates in *Cr-ecp* regulation, consistent with its broad role in silencing horizontally acquired and core genes through nucleation, spreading, and bridging along DNA (Navarre 2010; Rashid and Dame 2024). H-NS directly represses *Cr-ecp* promoter activity, as evidenced by both genetic (Fig. 5A) and biochemical (Fig. 5C) data. However, the inability of the Δ*hns* Δ*ihfA* double mutant to activate *Cr-ecp* expression (Fig. 5A) demonstrates that removal of H-NS is not sufficient to drive transcription. Therefore, *Cr-ecp* regulation involves at least two separable layers: H-NS-mediated repression and an IHF-dependent activation mechanism that requires the DRE.

The environmental conditions required for *Cr-ecp* activation also offer clues to its physiological relevance. *Cr-ecp* expression was maximal under static growth in DMEM at temperatures below 37 °C and was essentially undetectable in rich medium (LB) or at 37 °C, a pattern broadly consistent with the temperature- and medium-dependent regulation described for *ecp* in EPEC and EHEC (Lehti et al. 2013; Martinez-Santos et al. 2012). While we are cautious in extrapolating from these laboratory conditions to the intestinal environment, the observed downregulation at 37 °C is notable given that *C. rodentium* colonizes the mammalian gut at host body temperature. This raises the possibility that *Cr*-*ecp* is not primarily activated during intestinal colonization itself, but rather during phases of the infection cycle associated with lower environmental temperatures, such as fecal-oral transmission between hosts (Collins et al. 2014; Mullineaux-Sanders et al. 2019). Consistent with this idea, ECP has been shown to mediate EHEC attachment to spinach leaf surfaces (Rossez et al. 2014; Saldana et al. 2011), a plant-associated transmission route in which the bacterium persists at temperatures well below 37 °C. Under this scenario, ECP could contribute to bacterial persistence or attachment outside the mammalian host, or during the initial stages of colonization prior to full adaptation to host body temperature. Importantly, the specific physiological and environmental conditions under which the DRE-mediated regulatory mechanism operates during natural infection or host-to-host transmission remain unknown, and defining the relevance of the DRE and its associated regulatory circuit under these conditions will be an important direction for future work.

This study defines the *cis*-regulatory and NAP-dependent organization of the *Cr-ecp* promoter but also establishes the limitations of the current model. A central open question raised by this work is the identity of the trans-acting factor that recognizes the DRE, which remains to be identified. Second, formal demonstration of binding specificity and identification of the precise binding sites and DNA architecture at the promoter will require footprinting and additional mutational analyses. In support of the EMSA data presented here, in silico analysis identified several candidate IHF- and H-NS-binding sites within the *Cr-ecp* regulatory region (Fig. S1). Whether these predicted sites correspond to the functional contacts made by IHF and H-NS in vivo, however, remains to be experimentally determined. Third, the dispensability of *C. rodentium* EcpR under the conditions tested does not exclude a role for this regulator under other environmental or host-associated conditions, nor a potential post-transcriptional contribution as previously suggested for *E. coli* MatA (EcpR) (Lehti et al. 2013). In *E. coli*, in addition to EcpR, *ecp* regulation also involves a network of regulators including IHF, H-NS, the response regulator RcsB of the Rcs phosphorelay (Lehti et al. 2012a, b; Martinez-Santos et al. 2012; Pannen et al. 2016), and the RNA-binding protein Hfq (Pearl Mizrahi et al. 2021). Notably, in *E. coli* the distal regulatory region contains an RcsB box (Wehland and Bernhard 2000) that overlaps the EcpR-binding TTCCT box (Martinez-Santos et al. 2012) and is essential for RcsB-dependent activation of the *mat* operon (Lehti et al. 2012a, b); the 10-bp core of the *Cr-ecp* DRE (TAAGAATATC) corresponds closely to the first half of this RcsB consensus (Fig. S1). The absence in *Cr-ecp* of the EcpR-binding TTCCT boxes, combined with the conservation of a sequence motif resembling the RcsB box, raises the question of which of these regulatory inputs are conserved, lost, or replaced in *C. rodentium*. Whether the trans-acting factor binding the *Cr-ecp* DRE corresponds to RcsB, a related response regulator, or a distinct factor altogether, and whether it operates alone or in combination with an additional partner, given the well-documented promiscuity of RcsB in forming heterodimers with diverse coregulators (Pannen et al. 2016), will be a matter of future investigation, essential for understanding how *Cr-ecp* expression is integrated with environmental cues.

In summary, this work defines a regulatory framework for *Cr-ecp* in which a distal *cis*-acting element (DRE) and the nucleoid-associated proteins IHF and H-NS cooperate to control promoter activity, with the strictly positive role of IHF distinguishing this system from the EPEC and EHEC models. This framework retains some regulatory principles of *E. coli*, including control by NAPs and distal regulatory information, but differs in its independence from EcpR-mediated activation. More broadly, these findings illustrate how conserved fimbrial systems can maintain related structural organization while evolving distinct regulatory strategies, underscoring the importance of comparative analyses for understanding how species-specific environmental cues shape regulatory networks.

## Supplementary Information

Below is the link to the electronic supplementary material.

Supplementary Material 1.

Supplementary Material 2.

Supplementary Material 3.

Supplementary Material 4.

## Data Availability

The datasets generated and analyzed during the current study are available from the corresponding author upon reasonable request.
