# Supplementary material for: Contrasting regulation of the ecp common pilus operon in Citrobacter rodentium: essential roles of a distal element and IHF even in the absence of H-NS
Source: Arch Microbiol. 2026 Sep 21;208(12):658. doi: 10.1007/s00203-026-05216-7 (PMC13593695; doi:10.1007/s00203-026-05216-7)
Supplement: Supplementary file 1 [file 203_2026_5216_MOESM3_ESM.docx]

**Tabla S1 | Strains and plasmids used in this study.**

|  | **Description** | **Antibiotic resistance** | **Reference** |
| --- | --- | --- | --- |
| **Strains** |  |  |  |
| *E. coli* DH5-α | λ^-^,Φ80 *lacZ*ΔM15 Δ(*lacZYAargF*) U169 *rec*A1 *end*A1 *hsd*R17 (r_K_^-^, m_K_^+^) *pho*A *sup*E44 λ^–^ *thi*-1 *gyr*A96 *rel*A1 | Nal | Invitrogen |
| DBS100 | Wild type *C. rodentium* | No | (Schauer and Falkow 1993) |
| JPCR15 | *ecpA*::3XFLAG-Km, DBS100 derivative expressing EcpA-3XFLAG | Km | This study |
| JPCR17 | *ecpR*::3XFLAG-Km, DBS100 derivative expressing EcpR-3XFLAG | Km | This study |
| JPCR20 | Δ*ecpR*::Km, DBS100 derivative with deletion of the *ecpR* gene | Km | This study |
| JPCR13 | Δ*hns,* JPCR1 derivative upon excision of the Km cassette | No | (Caballero-Flores et al. 2015) |
| JPCR21 | Δ*ihfA*::Km, DBS100 derivative with deletion of the *ihfA* gene | Km | This study |
| JPCR25 | Δ*ihfA*::Km Δ*hns,* Δ*hns* derivative with deletion of the *ihfA* gene | Km | This study |
| JPCR26 | Δ*ecpR* *ecpA*::3XFLAG-Km, Δ*ecpR*::Km derivative upon excision of the Km cassette, expressing EcpA-FLAG | Km | This study |
| **Plasmids** |  |  |  |
| pKD46 | λ Red recombinase system under the control of the araBAD promoter | Ap | (Datsenko and Wanner 2000) |
| pKD3 | Chloramphenicol cassette template plasmid | Ap Cm | (Datsenko and Wanner 2000) |
| pKD4 | Kanamycin cassette template plasmid | Ap Km | (Datsenko and Wanner 2000) |
| pSUB11 | 3XFLAG epitope template plasmid | Ap Km | (Uzzau et al. 2001) |
| pCP20 | FLP recombinase plasmid | Ap | (Datsenko and Wanner 2000) |
| pMPM-T3 | Low-copy-number cloning vector; p15A derivative | Tc | (Mayer 1995) |
| pT3-HNS | pMPM-T3 derivative carrying the *hns* gene | Tc | (Bustamante et al. 2001) |
| pT3-*ihfA* | pMPM-T3 derivative carrying the *ihfA* gene of EPEC E2348/69 | Tc | This study |
| pMD-HNS-MH | pBAD derivative expressing *Salmonella enterica* serovar Typhi H-NS-Myc/His | Ap | (Banda et al. 2018) |
| pKK-232-8 | pBR322 derivative containing a promoterless chloramphenicol acetyltransferase (*cat*) reporter gene | Ap | Pharmacia Biotech |
| p*ecpA*-1 | *ecpA*-*cat* transcriptional fusion from position -494 to +211^a^ | Ap | This study |
| p*ecpA*-2 | p*ecpA*-1 derivative with a modified -10 promoter sequence (GATAAT to CGCCGT) | Ap | This study |
| p*ecpA*-3 | *ecpA*-*cat* transcriptional fusion from position -494 to +98^a^ | Ap | This study |
| p*ecpA*-4 | *ecpA*-*cat* transcriptional fusion from position -409 to +211^a^ | Ap | This study |
| p*ecpA*-4.1 | *ecpA*-*cat* transcriptional fusion from position -383 to +211^a^ | Ap | This study |
| p*ecpA*-DRE1 | *ecpA*-*cat* transcriptional fusion from position -383 to +211 with a modified DRE1 sequence (CGCGG to ATATT)^a^ | Ap | This study |
| p*ecpA*-DRE2 | *ecpA*-*cat* transcriptional fusion from position -383 to +211 with a modified DRE2 sequence (AAAAT to CCCCG)^a^ | Ap | This study |
| p*ecpA*-DRE3 | *ecpA*-*cat* transcriptional fusion from position -383 to +211 with a modified DRE3 sequence (TAAGA to GCCTC)^a^ | Ap | This study |
| p*ecpA*-DRE4 | *ecpA*-*cat* transcriptional fusion from position -383 to +211 with a modified DRE4 sequence (ATATC to CGCGA)^a^ | Ap | This study |
| p*ecpA*-DRE5 | *ecpA*-*cat* transcriptional fusion from position -383 to +211 with a modified DRE5 sequence (CCTGA to AAGTC)^a^ | Ap | This study |
| p*ecpA*-DRE6 | *ecpA*-*cat* transcriptional fusion from position -383 to +211 with a modified DRE6 sequence (AATTA to CCGGC)^a^ | Ap | This study |
| p*ecpA*-4.2 | *ecpA*-*cat* transcriptional fusion from position -357 to +211^a^ | Ap | This study |
| p*ecpA*-5 | *ecpA*-*cat* transcriptional fusion from position -334 to +211^a^ | Ap | This study |
| p*ecpA*-6 | *ecpA*-*cat* transcriptional fusion from position -211 to +211^a^ | Ap | This study |
| p*ecpA*-7 | *ecpA*-*cat* transcriptional fusion from position -127 to +211^a^ | Ap | This study |
| p*ecpA*-8 | *ecpA*-*cat* transcriptional fusion from position -20 to +211^a^ | Ap | This study |
| p*ecpA*-9 | *ecpA*-*cat* transcriptional fusion from position +34 to +211^a^ | Ap | This study |
| p*ecpA*-10 | *ecpA-cat* transcriptional fusion from position -494 to -28^a^ | Ap | This study |

^a^ Coordinates of the fragments contained in the transcriptional fusions are with respect to the transcriptional start site (TSS) of the *ecp* operon.

**Tabla S2 | Oligonucleotides used in this study.**

| **Name** | **Sequence** |
| --- | --- |
| **Tagged and mutant strains** | |
| cat-1 | TTATACGCAAGGCGACAAGG |
| cat-2 | GATCTTCCGTCACAGGTAGG |
| k1 | CAGTCATAGCCGAATAGCCT |
| k2 | CGGTGCCCTGAATGAACTGC |
| Cr-ecpA-P1 | GTTCAGTTTACCGCTACATGGACCACGCCGGCAGCAACGCCGGACTACAAAGACCATGACGG^a^ |
| Cr-ecpA-P2 | GAAAATTCGGGAGAAATGCCTCCCGAATCATTATCATCACGTCATATGAATATCCTCCTTAG^a^ |
| Cr-ecpA-F2 | AACAAAAACCTCCGGTCTGG |
| Cr-ecpR-P1 | CGGCTTGAAAAACTCATTATTGAGCACTTCCATAGCCGATGCGACTACAAAGACCATGACGG^a^ |
| Cr-ecpR-P2 | CCCGCTTTCGATGTCGATGCAAACAGGTCAGAAAACGTTGCGCATATGAATATCCTCCTTAG^a^ |
| Cr-ecpR-F2 | TTAACGGGAAAAGCATTATGC |
| Cr-ecpR-H1 | CGGCAAAGGGGGCGGCTCATGAATTTTACATTACCGATGAATTGTAGGCTGGAGCTGCTTCG^a^ |
| Cr-ecpR-H2 | AGGTCAGAAAACGTTGCGTCAGCATCGGCTATGGAAGTGCTCCATATGAATATCCTCCTTAG^a^ |
| Cr-ecpR-R | TGTCAACAGCAATGGTTAGTC |
| CrhimA-H1 | CATCATTGAGGGATTGAACCTATGGCGCTTACAAAAGCTGAATGTAGGCTGGAGCTGCTTCG^a^ |
| CrhimA-H2B | TTAGTCAGGTTTGGATCAAATTACTCATCTTTGGGCGAAGCGTTTTCAACCCGGCTTTTTAACATATGAATATCCTCCTTAG^a^ |
| CrhimA-F | CGTAAATCAGGTAGTTGGCGT |
| himA-RvMIIC | CCGTTTGATACGCCGAGTAA |
| **Plasmids** |  |
| pMPMT3/K3 Fw | TGTGGAATTGTGAGCGGATAAC |
| pMPMT3/K3 Rv | AAGGCGATTAAGTTGGGTAACG |
| ihfA-KpnF | CGATTCCAGGTACCATTGAGGG^b^ |
| ihfA-XhoR | GCCTCTCGAGTTAGATCAGATTACTC^b^ |
| **Transcriptional fusions** |  |
| pKK-SEQ2-F | GAGGCCCTTTCGTCTTCAAG |
| pKK-SEQ1-R | CAACGGTGGTATATCCAGTG |
| pkk-8-BH1F | GGAATTCTCGGGGAT |
| pkk-8-H3R | GTTTTGTCCTACTCAAGC |
| CrecpAF-582 | GTTTTCAGGGATCCCTCATCTCAC^b^ |
| D-5a10Fw | TCAAAATCGCCGTTTTACTGCGGCAATT |
| D-5a10Rv | GTAAAACGGCGATTTTGAAAAATCGGAGC |
| EcpABH1F-497 | AAATGAGTTGGATCCGACGGATAG^b^ |
| EcpABH1Fw-383 | CGCGACATTGGATCCCGCGGAAAATTA^b^ |
| EcpABH1Fw-357 | ATCGGATCCATTAGCCTTTTATTATAG^b^ |
| EcpABH1F-422 | TATTATAGGATCCTTCCCCAACAAC^b^ |
| EcpABH1F-299 | GTTGTGAGATGGATCCTAGAGTTTT^b^ |
| EcpABH1F-215 | CCAAATCCAGGATCCCAACCAAAC^b^ |
| EcpABH1F-113 | TGAGCGCTCGGATCCTTCAAAAGA^b^ |
| EcpABH1F-55 | ATAAAAAAGGATCCGCGCGCG^b^ |
| EcpA-ERD-1F | CCCGGGGATCCATATTAAAATTAAGAATATCCCT^b^ |
| EcpA-ERD-2F | CCCGGGGATCCCGCGGCCCCGTAAGAATATCCCTGAAAT^b^ |
| EcpA-ERD-3F | CCCGGGGATCCCGCGGAAAATGCCTCATATCCCTGAAATTAGCC^b^ |
| EcpA-ERD-4F | CCCGGGGATCCCGCGGAAAATTAAGACGCGACCTGAAATTAGCCTTTTA^b^ |
| EcpA-ERD-5F | CCCGGGGATCCCGCGGAAAATTAAGAATATCAAGTCAATTAGCCTTTTATTAT^b^ |
| EcpA-ERD-6F | CCCGGGGATCCCGCGGAAAATTAAGAATATCCCTGACCGGCGCCTTTTATTATAGGATT^b^ |
| CrecpA-H3R | GTAATAACAAGCTTGCTTCTGGTG^b^ |
| ecpAHdR+10 | GCGGCCAGTAAGCTTTTTTTCAC^b^ |
|  |  |
| **Fragments for EMSA** |  |
| f17A_pBAD_F | CACCCATGGTCACGCAGGGTACCGTAAC^b^ |
| f17A_pBAD_R CGGAAGCTTCGGATACGCGATGGTATAAGCC^b^ | |
|  |  |

^a^ Underlining indicates homologous region to template plasmid pKD4 for allelic reemplace or to plasmid pSUB11 for tagging.

^b^ Underlining indicates the introduced restriction site.
