# Supplementary material for: Contrasting regulation of the ecp common pilus operon in Citrobacter rodentium: essential roles of a distal element and IHF even in the absence of H-NS
Source: Arch Microbiol. 2026 Sep 21;208(12):658. doi: 10.1007/s00203-026-05216-7 (PMC13593695; doi:10.1007/s00203-026-05216-7)
Supplement: Supplementary file 2 [file 203_2026_5216_MOESM4_ESM.docx]

**Figure S1. Sequence alignment of the *ecp* upstream regulatory regions of *E. coli* and *C. rodentium*.**

Alignment was generated with Clustal Omega using sequences from *C. rodentium* ICC168 and EPEC E2348/69 retrieved from KEGG. The TTCCT boxes of *E. coli ecp* (Martínez-Santos et al., 2012) and the DRE core sequence (TAAGAATATC) of *Cr-ecp* are highlighted in red bold letters. DNA motifs resembling the RcsB consensus binding sequence are indicated by purple boxes. Candidate IHF-binding sites, identified using the Virtual Footprint platform with the *E. coli* IHF position weight matrix, are highlighted in yellow (underlined, negative strand) or green (positive strand). Only sites with the highest relative score (0.90–0.95) are shown. Candidate H-NS-binding sites, identified using the same platform, are highlighted in turquoise (negative strand; score 0.96-0-86). The transcription start sites (TSS) and predicted −35 and −10 hexamers are in bold and underlined.

**Figure S2. Conservation of the DRE core among *Citrobacter* species.**

Multiple sequence alignment of the *ecpA* upstream regulatory region across eight *Citrobacter* species, generated with Clustal Omega using orthologous sequences retrieved from KEGG (see Materials and Methods, section 2.11, for strain and entry identifiers). For each species, the 57-bp region encompassing the DRE and its immediate flanking sequence is shown. In *C. rodentium*, the DRE (subdivided into DRE1–DRE6, as defined in **Fig. 4A**) is indicated in bold. Asterisks below the alignment denote columns of full sequence identity across all species compared; dots denote positions conserved in all but one sequence.
